# A general health promotion approach to helping smokers with non-communicable diseases quit smoking: A pilot randomized controlled trial

**DOI:** 10.3389/fpubh.2022.957547

**Published:** 2022-10-18

**Authors:** William Ho Cheung Li, Laurie Long Kwan Ho, Ankie Tan Cheung, Man Ping Wong, Derek Yee Tak Cheung, Wei Xia, Tai Hing Lam

**Affiliations:** ^1^The Nethersole School of Nursing, The Chinese University of Hong Kong, Hong Kong, Hong Kong SAR, China; ^2^Li Ka Shing Faculty of Medicine, School of Nursing, The University of Hong Kong, Hong Kong, Hong Kong SAR, China; ^3^School of Nursing, The Sun Yat-sen University of Medical Sciences, Guangzhou, China; ^4^Li Ka Shing Faculty of Medicine, School of Public Health, The University of Hong Kong, Hong Kong, Hong Kong SAR, China

**Keywords:** mobile technology, information communication technology, non-communicable disease, smoking cessation, brief motivational interviewing

## Abstract

**Background:**

Despite evidence showing that continued smoking in patients with non-communicable diseases can reduce treatment efficacy and increase the risk of disease progression and multimorbidity, many smoker patients either have no intention to quit or have had failed attempts at quitting.

**Objective:**

To examine the feasibility of a general health promotion approach that uses instant messaging to deliver brief motivational interviewing to help smokers with non-communicable diseases quit smoking.

**Methods:**

In total, 60 participants who had medical follow-up in a special out-patient clinic were randomized into two groups, 30 in the intervention group received brief motivational interviewing to assist them with their chosen behavioral changes, and 30 in the control group received only a smoking cessation booklet. The outcome measures included self-reported 7-day point prevalence of smoking abstinence and any behavioral change reported by the participants at 6 and 12 months. Biochemical validation was performed for those who verbally reported a 7-day point prevalence of smoking abstinence at 12 months.

**Results:**

The majority (95%) of smokers who attended the out-patient clinic owned a smartphone. The response rate was 73.2%. Retention rates at 6-month and 12-month follow-up were 83.3 and 71.7%, respectively. The process evaluation indicated that participants were satisfied with the content of the brief MI messages and appreciated the use of instant messaging as a way to provide them with professional advice and support for managing their health-related lifestyles. The intervention group had a higher biochemically validated abstinence rate than the control group at 12 months (16.7 vs. 6.7 *P* = 0.23) although the difference was not statistically significant (Adjusted odd ratio 2.4, 95% confidence interval, 0.43–13.75; *P* = 0.32.), In addition, the proportion of participants reporting a behavioral change was higher in the intervention group at 6 and 12 months.

**Conclusion:**

This study suggested the potential efficacy and feasibility of a general health promotion approach that uses instant messaging to deliver brief motivational interviewing to help smokers with non-communicable diseases quit smoking. The findings can be used to create a new smoking cessation service model that implements a flexible, proactive and personalized approach to help smokers quit smoking.

**Clinical trial registration:**

ClinicalTrials.gov, identifier: NCT03983330.

## Introduction

Smoking plays a causal role in the development of non-communicable diseases (NCDs), including cardiovascular disease, chronic respiratory disease, cancer and diabetes (1). According to the World Health Organization (2), 14% of all NCD-related deaths worldwide are attributable to smoking. There is evidence that continued smoking by patients with NCDs can reduce their treatment efficacy and increase the risk of side effects (3, 4), whereas quitting after diagnosis can reduce the risk of disease progression, ameliorate adverse treatment-related effects, and improve disease prognosis and quality of life (5, 6).

Over the years, the Hong Kong government and community have put enormous efforts on raising tobacco tax, legislation, law enforcement and health promotion and provision of smoking cessation services. The prevalence of daily cigarette smokers has been decreasing from 23.3% in 1982 to 9.5 % in 2021 (7). Despite a significant fall in the prevalence of smoking over the past few decades, the rate of decline in smoking prevalence has slowed down and may not be sustainable as a result of the existence of a group of chronic smokers. The group of chronic smokers represents a challenge because they are less likely to be affected by the current tobacco control interventions or policies, eventually will make up a larger proportion of the remaining smokers in the community. Smokers with NCDs are often chronic smokers with a long smoking history and high nicotine dependency. These patients may thus have no intention of quitting or have had failed attempts at quitting (6, 8, 9). Previous smoking cessation trials have shown that ~73% of smokers with cancer (9), 68% with cardiac diseases (10), and 70% with diabetes mellitus (11) have no intention of quitting even smoking cessation interventions such as stage-matched interventions, brief advice based on risk communication, individualized face-to-face counseling by trained nurses were given (9–11). Thus, there is an urgent need for an innovative and effective smoking cessation intervention targeted at this group of smokers.

Smoking is associated with physical inactivity, an unhealthy diet and alcohol consumption (12, 13). This interrelationship suggests that there may be a higher-level attribute that determines all such health behaviors. Previous studies have shown that individuals with a general intention to improve their health are more likely to engage in desirable health-related lifestyle practices, and once engaged, will progress to other healthy lifestyle practices (14, 15). Moreover, a large body of evidence supports the effectiveness of brief intervention for smoking cessation, such as brief behavior change counseling and opportunistic advice (16–18). Based on this concept, a general health promotion approach using brief motivational interviewing (MI) may benefit patients with NCDs who smoke by motivating them to first choose and then engage in any desirable health-related lifestyle practice, such as exercising regularly or maintaining a healthy diet, and then eventually to quit smoking. However, rigorous empirical scrutiny of the efficacy of such an approach has not been conducted.

The World Health Organization defines medical and public health practice supported by mobile devices as mobile health, a new strategy to promote health (19). Instant messaging, such as WhatsApp/WeChat delivered by mobile devices, is widely used for health promotion and treatment compliance (20). One advantage of using WhatsApp/WeChat is that it can offer quick, real-time interactions and continuing professional advice and support for subjects to manage their health-related lifestyle practices. Most importantly, WhatsApp/WeChat is more flexible, efficient and time-saving compared to face-to-face meetings to deliver a brief MI as face-to-face meetings would require the subjects to return several times for interventions. A meta-analysis of the use of mobile phone-based interventions for smoking cessation showed that smokers who received instant messages via mobile phones were ~1.7 times more likely to abstain from smoking than those who received conventional face-to-face cessation services (21).

This study thus aimed to examine the feasibility of a general health promotion approach that uses instant messaging to deliver brief MI to help smokers with NCDs quit smoking, in term of recruitment process, participation rate, retention rate, and appropriateness of assessment tools and the content of the intervention.

### Theoretical framework

The proposed intervention was guided by the brief MI. MI differs from prevailing patient education methods. It is a directive, client-centered counseling strategy that encourages clients to explore and resolve their ambivalences and promotes their confidence in their ability to change their behavior. It involves eliciting an individual's motivation, enhancing his or her commitment and exploring solutions that can promote behavioral change in four stages: engaging, focusing, evoking and planning (22). Brief MI shares the same core as MI: interventionists function as advocates to help individuals, often in a state of ambivalence with fluctuating motivation, to initiate and continue behavioral change (23). Thus, both brief MI and MI focus on using specific techniques to explore and resolve individuals' doubts and develop discrepancies between their core beliefs and their behavior of not engaging in desirable health-related lifestyle practices, consequently enhancing their confidence in engaging and motivation to engage in behavioral change. Because MI generally takes more than 30 min to implement and is not feasible in busy clinical settings, brief MI has been developed for brief consultations in such settings (23). Brief MI adopts shorter and simpler strategies, which usually lasts for 5–10 min (23).

## Methods

### Study design

A single-blind, pilot RCT was conducted in a Special Out-Patient Clinic (SOPC) in one of the largest acute care hospitals in Hong Kong. This study followed the Consolidated Standards of Reporting Trials (CONSORT) 2010 guideline. The study was registered in the Registration ClinicalTrials.gov Identifier: NCT03983330 and approval by the Institutional Review Board of the University of Hong Kong and Hospital Authority Hong Kong West Cluster (Reference, UW19-117). The research protocol has been published elsewhere (13). To ensure that participants' right were protected, the researchers strictly adhered to the Declaration of Helsinki and ethical principles in designing and conducting the research.

### Participants

Hong Kong Chinese smokers with NCDs who had medical follow-up in the SOPC and met the following inclusion criteria were invited to participate: (1) aged 18 years or above, (2) able to speak Cantonese and read Chinese, (3) having no intention of quitting but willing to take other steps to improve health, (4) owning a smartphone and able to use instant messaging (e.g., WhatsApp and WeChat) for communication, and (5) willing to receive health promotion advice via WhatsApp/WeChat on the smartphone throughout the study period. Potential participants were excluded if they were (1) unable to provide informed consent or participate in our intervention due to impaired mental status, cognitive impairment, or communication barrier, and were (2) currently participating in or accessing another smoking cessation program or service. A self-help booklet on smoking cessation and brief smoking cessation advice were given to those excluded smokers with NCDs who had the intention to quit or who did not use a smartphone or an instant messaging application. They were also referred to the other existing smoking cessation programs and services if they consented.

We have found no similar intervention in the literature. However, a previous literature suggests that at least 12 subjects per group would be needed for a pilot study (24). A significance level of 5% (2-tailed) and to account for a potential attrition rate of 30% at the 12-month follow-up assessment, totally at least 34 participants will be recruited. However, with the available resources and the proposed timeframe, we will be able to recruit 60 subjects. We therefore propose to have the sample size of 60 for this feasibility study, with 30 in the experimental group and 30 in the control group.

### Randomization

Sequentially numbered opaque sealed envelopes (SNOSEs) and block randomization were used to individually allocate the participants to the control or intervention group such that both groups contained equal numbers of participants. Block of six in random order was created with equal numbers to be allocated to the two groups. The envelopes were labeled with serial numbers, and the interventionists were blinded to the random allocation sequence. Once a smoker signed the consent form, the interventionist opened a SNOSE according to the participant's serial number to determine the group allocation. To ensure privacy and prevent the possibility of interaction between the two groups in the same setting (i.e., to minimize contamination), the baseline assessment and intervention for each participant were conducted in a single room. The data analysts were blinded to the group assignments.

### Interventions

#### Intervention group

Participants in this group received a general health promotion approach that uses instant messaging to deliver brief MI to help smokers with NCDs quit smoking. Introduced by Freedman and Fraser (25), the general health approach adopted the foot-in-the-door technique that emphasizes the notion that individuals who are induced to comply with a smaller and easier request initially are more likely to comply later with a larger request (25). Agreement to the first request or target increases the individuals' confidence and alters their self-perceived capability and willingness to comply with further requests or targets.

In the SOPC, the participants were first asked to fill in a baseline questionnaire. Using a general health approach, a trained research nurse then asked each participant about his/her priorities for engaging in any desirable health-related lifestyle practice as identified in the completed baseline questionnaire. The participants were also asked to choose a goal that they considered easiest to achieve, such as eating more vegetables or less fried food, doing more exercise or reducing alcohol consumption. Each participant then received an individual face-to-face brief MI session (~5 min) that provided generic health advice on the selected health-related lifestyle practice. At the end of the face-to-face session, each participant was informed that throughout the study period, the interventionist (trained research nurse) would help him/her achieve the chosen health-related goal through motivational messages via WeChat or WhatsApp. The participants were also given a self-help booklet on smoking cessation.

For the first 6 months of the study period, the trained nurse delivered brief MI messages more or less intensively based on the participant's responses (usually not less than once per 2 or 3 days and no more than twice per day). The participants could have several chat sessions with the trained nurse over several days/weeks, but not exceeding the total amount of time that would be spent over fewer, longer sessions of traditional MI. After 6 months until 1-year follow-up, the trained nurse sent minimal messages, merely to follow the participants' progress, respond to their questions and maintain contact.

**Figure 1:**
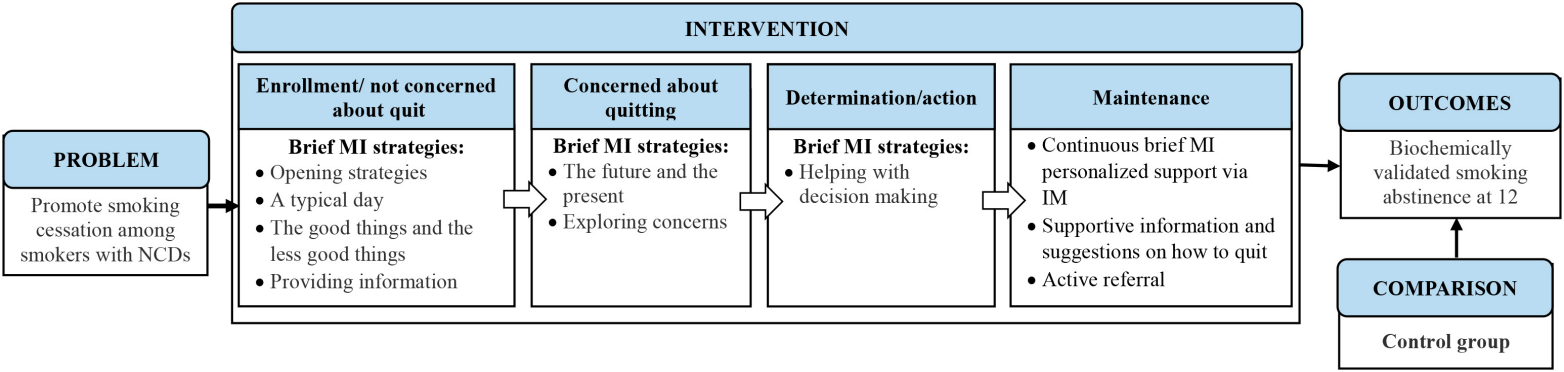
Conceptual framework.

The content of the brief MI messages depended on the desirable health-related lifestyle practice and the targeted goal that each participant perceived to be the easiest to achieve. The aim of the messages was to help the participant move toward his/her goal. Messages on the current lifestyle practice were guided by the framework (Figure 1) and the list of strategies (Table 1). The brief MI messages were individualized and specific to participants' choice of lifestyle practice and their progress of change. For the nurse to move down the menu, the participant had to show greater readiness to change. The trained nurse implemented the technique of expressing empathy, a learnable skill for understanding another individual's meaning through the use of reflective listening (22), in the messages. She was reminded not to direct or judge the participant's decisions, and instead show an attitude of acceptance, but not necessarily approval or agreement, and to express that ambivalence was normal (22).

**Table 1:** The menu of strategies in brief motivational interviewing.

**Strategies**	**Aims**	**Examples of questions**
1. Opening strategy: lifestyle, stress and current smoking habit	To establish rapport and understand the context of the smoking habit	‘What is your smoking status now?' ‘Can you tell me more about your smoking habit?'
2. Opening strategy: health and current smoking habit	To build rapport and relate health problems to the smoking habit	‘How does your smoking habit affect your health?' ‘How does your smoking habit relate to your current health condition?'
3. A typical day	To further establish rapport, to assist the participant in discussing the current smoking habit in detail and to assess the level of readiness to quit	‘Can you tell me about your smoking habit in a day from the beginning to the end?' ‘Can you describe your smoking habit in a typical day, like yesterday: when did you have your first cigarette, and how frequently did you smoke until the end of the day? May we start from the beginning?'
4. The good things and the less good things	To explore participant's feelings about not quitting smoking.	‘What are the pros and cons of not quitting smoking?' ‘What are your barriers to quitting/your perceived benefits of quitting?' ‘What do you like/dislike about your smoking habit?'
5. Providing information	To provide relevant information on quitting smoking in a sensitive manner	‘I wonder if you would be interested in knowing the health benefits of quitting smoking'. ‘I wonder if you would like to know more about how to overcome withdrawal symptoms or cigarette cravings.' ‘I wonder what you would do after knowing the health benefits of quitting'. ‘How do these relate to changing your smoking habit?'
6. The future and the present	To shed light on discrepancies, explore concerns about quitting and motivate participants to think about quitting smoking	‘How would you like your smoking habit to be in the future (e.g. quitting smoking)?', ‘What is your major barrier to quitting smoking?'
7. Exploring concerns	To help participants identify and explore their concerns about quitting smoking	‘What concerns do you have about engaging in quitting smoking?' ‘What other concerns do you have now?' ‘What else, what other concerns do you have?'
8. Helping with decision-making	To assist participants in decision-making to quit smoking	‘Where does this leave you now?', ‘What is your plan for quitting smoking in the coming months?', ‘What behavioural changes are you going to implement now?'

##### Assessment of readiness to quit at 3-month follow-up

Although the participants did not have an intention to quit smoking at baseline, readiness to quit smoking was assessed at 3-month follow-up. For participants who were willing to take further action to improve their health, i.e., who had developed an intention to quit smoking, brief MI messages on smoking cessation guided by the menu of strategies were delivered by the nurse. The participants were provided with more comprehensive information on quitting upon their request. At this stage of the intervention, the skills needed to overcome nicotine withdrawal symptoms and cravings were discussed. The participants could select their own schedules for quitting (quitting immediately or progressively). A previous study showed that giving smokers the choice to select their own schedules for quitting tends to enhance their self-efficacy to quit smoking (26).

##### Fidelity of intervention implementation

A registered nurse was employed as the trained research nurse to deliver the proposed interventions to the participants. Before commencing the study, the research nurse and the research team received a two half-day brief MI training workshop conducted by a clinical psychologist (trainer) via demonstrations, reciprocation and role play. The workshop content included knowledge and skills on the principles and practices of brief MI. The trainer had extensive experience in using brief MI to motivate behavioral change. After the workshop, to ensure the trained nurse's quality and competence in applying the learned skills through instant messaging, she was asked to complete an assessment delivered by the trainer that involved a case study examination. The nurse was asked to strictly follow the intervention protocol. The research team periodically checked the recruitment of participants as well as the delivery of the intervention by the nurse by reviewing the messages sent by her through WhatsApp or WeChat. The research team met with the nurse every other week to evaluate the quality of the intervention implementation. All of the brief MI sessions were digitally recorded for review, but the participants' personal data were kept confidential and anonymous.

#### Control group

Similar to the intervention group, participants in the control group received a self-help smoking cessation booklet at baseline that provided information on the consequences of smoking and benefits of smoking cessation, simple strategies for managing withdrawal symptoms, introduction to available smoking cessation aids, and information about common smoking cessation services. They were asked by the research nurse about their priorities for engaging in any desirable health-related lifestyle practice and to state a targeted goal that they perceived as the easiest to achieve after completing the baseline questionnaire. These participants did not receive brief MI or booster intervention, but did receive follow-up telephone calls at 1, 3, 6 and 12 months, as in the intervention group.

### Outcome measures

#### Baseline measures

Participants' baseline data, including demographic characteristics, health status and smoking history, were obtained using a structured questionnaire based on previous trials (9–11). The questionnaire was administered face-to-face by a trained research nurse before randomization.

Follow-up visits were conducted at 1, 3, 6, and 12 months in accordance with the guidelines of the Society for Research on Nicotine and Tobacco (27). The outcome measures included (i) self-reported 7-day point prevalence of smoking abstinence at 6 and 12 months, and (ii) any behavioral change reported by the participants at 6 and 12 months. Biochemical validation was performed for those who verbally reported a 7-day point prevalence of smoking abstinence at 12 months, with the guidance of the trained research nurse in a private room or at venues appropriate to the participants. This involved measuring the carbon monoxide level in the participant's expired air and the cotinine levels in their saliva in a parallel test; a carbon monoxide level of < 4 ppm in the expired air and a salivary cotinine level of less than 115 ng/mL (28) were considered to be in good agreement with self-reported 7-day point prevalence of abstinence (29, 30). Only the participants who passed the validation test were regarded as having biochemically validated abstinence. For behavioral change, participants were also asked to self-report any changes on their selected health-related lifestyle practice and assess whether they achieved the chosen health-related goal at the baseline.

For assessing the feasibility of the study, recruitment process, participation rate, retention rate will be documented. To the acceptability of the intervention, a process evaluation was conducted on all participants in the intervention group at 12-month follow-up visit. Participants were asked to comment on the appropriateness and comprehensiveness of the content and frequency of brief MI messages, and the assessment methods.

### Data analysis

Statistical analyses were conducted using IBM SPSS Statistics for Windows (version 25.0; IBM). Baseline characteristics of the intervention and control group participants were first compared using a chi-square test for categorical variables and an analysis of variance for continuous variables. An intention-to-treat analysis was performed by imputing all non-responses at follow-up by baseline values, i.e., assuming failures or no changes after intervention, to yield more conservative estimates of the effect size.

The analyses included the evaluation of the main efficacy of intervention vs. control in terms of biochemically validated abstinence rates at 12 months, and all other outcomes at 6 and 12 months using a generalized estimating equation model (GEE) to calculate the adjusted odds ratios (AORs) after adjusting for the baseline demographic and clinical characteristics that showed a significant difference. The self-reported behavior change in the intervention and control groups at 6 and 12 months using a chi-square test or a Fisher's exact test if there were 5 or fewer participants per cell. A 5% level of significance for the 2-sided tests was assumed.

## Results

**Figure 2:**
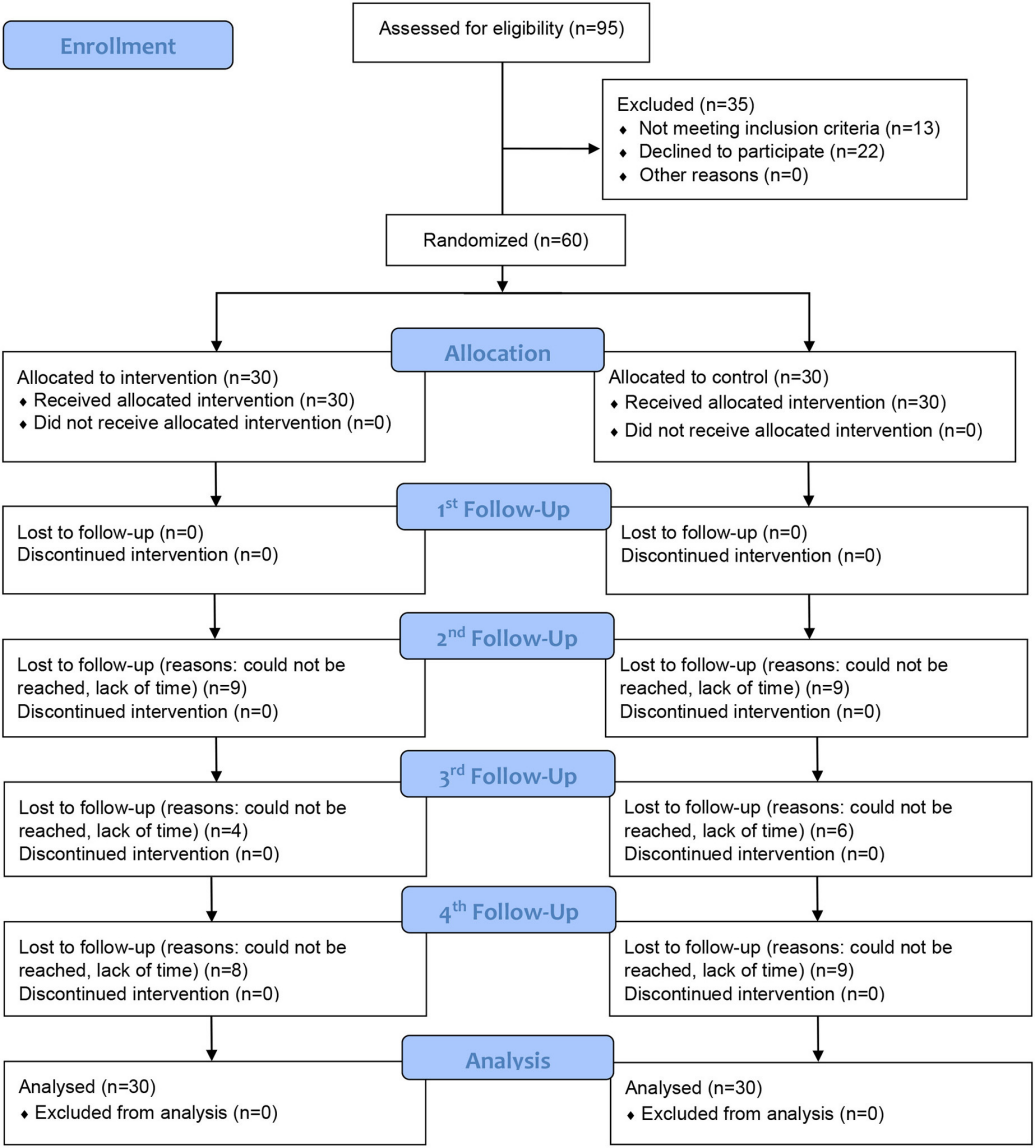
CONSORT flowchart.

Between 1 June 2019 and 17 July 2020, a total of 95 smokers with NCDs attended the SOPC were assessed for eligibility. Ninety-five percent of them owned a smartphone and could use instant messaging applications, and 82 (86.3%) were eligible for the study. Of these, 22 declined to participate, leaving 60 participants (a participation rate of 73.2%). We randomly assigned the participants into the intervention group (*n* = 30) receiving brief MI plus a self-help smoking cessation booklet or the control group (*n* = 30) receiving only a self-help smoking cessation booklet. All of the participants (100.0%) completed the 1-month follow-up, 42 (70%) completed the 3-month follow-up, 50 (83.3%) completed the 6-month follow-up, and 43 (71.7%) completed the 12-month follow-up. Figure 2 shows the CONSORT flowchart.

The demographic characteristics and smoking profiles of the participants are provided in Table 2. The participants included 51 men and 9 women with a mean [standard deviation (SD)] age at baseline of 46.2 (11.1) years. More than half of the participants (31 of 60) had multimorbidity. The mean (SD) years of smoking and daily cigarette consumption were 25.0 (11.0) and 13.5 (7.4), respectively. For the majority of the participants, nicotine dependency was moderate to severe (54 of 60) and no previous attempts to quit were reported (52 of 60). The intervention and control group participants had similar demographic characteristics and smoking profiles.

**Table 2:** Participants' demographic and smoking characteristics.

**Variable**	***N*** **(%)**		***P*** **-value**
	**Intervention group** **(*n* = 30)**	**Control group** **(*n* = 30)**	
Age, mean (SD), years	44.3 (10.2)	48.1 (12.0)	0.22
**Sex**			
Male	25(83.3)	26(86.7)	0.72
Female	5(16.7)	4(13.3)	
**Educational attainment**			
Primary or below	4(13.3)	5(16.7)	0.94
Secondary	22(73.3)	21(70.0)	
Tertiary	4(13.3)	4(13.3)	
**Marital status**			
Single	12(40.0)	11(36.7)	0.79
Married	18(60.0)	19(63.3)	
Employment status			
Employed	24(80.0)	21(70.0)	0.37
Unemployed or retired	6(20.0)	9(30.0)	
**Diagnosis**			
Cardiovascular diseases	6(20.0)	8(26.7)	0.64
Cancer	1(3.3)	0(0)	
Chronic respiratory diseases	6(20.0)	3(10.0)	
Diabetes	2(6.7)	3(10.0)	
Multiple chronic diseases	15(50.0)	16(53.5)	
Years of smoking, mean (SD), years	23.1 (9.7)	26.9 (12.3)	0.18
Daily cigarette consumption	13.5 (7.0)	13.5 (7.7)	0.97
**Nicotine dependency by the FTND**			
Mild, 0–3	2(6.7)	4(13.3)	0.54
Moderate, 4–5	10(33.3)	7(23.3)	
Severe, 6–10	18(60.0)	19(63.3)	
**Previous quit attempts**			
Yes	4(13.3)	4(13.3)	1.0
No	26(86.7)	26(86.7)	

FTND, Fagerström Test for Nicotine Dependence.

Table 3 shows the outcomes of the intervention and control group participants at 6 and 12 months. Biochemical validation was performed for 7 participants who self-reported abstinence at 12 months. Although the intervention group had a higher biochemically validated abstinence rate at 12 months than the control group, the difference was not statistically significant (16.7% [5 of 30] vs. 6.7% [2 of 30], *P* = 0.23). For intention to treat, the GEE model revealed an Adjusted odd ratio of 2.4 (95% confidence interval [CI], 0.43–13.75; *P* = 0.32) at 12 months in the intervention group after adjusting for age, sex, educational attainment, marital status, employment status, year of smoking and nicotine dependency. Complete case analysis was also conducted. The results showed that the difference in biochemically validated abstinence rate at 12 months between the intervention and control group was not statistically significant (22.7% [5 of 22] vs. 9.5% [2 of 21], *P* = 0.15). The odd ratio was 2.8 (95% confidence interval [CI], 0.48–14.33; *P* = 0.27) at 12 months in the intervention group after adjustment.

**Table 3:** The outcomes of participants in the intervention and control groups at 6 and 12 months.

**Variable**	***N*** **(%)**			**GEE model**			
	**Intervention group** **(*n* = 30)**	**Control group** **(*n* = 30)**	***P*** **-value**	**Crude ORs** **(95% CI)**	***P*** **-value**	**Adjusted ORs^a^** **(95% CI)**	***P*** **-value**
**Biochemically validated 7-day PPA**							
12 months	5 (16.7)	2 (6.7)	0.23	3.39 (0.57–20.10)	0.23	2.4 (0.43-13.75)	0.32
Self-reported 7-day PPA							
6 months	4 (13.3)	1 (3.3)	0.35	4.46 (0.47–42.51)	0.19	6.23 (0.62-62.94)	0.12
12 months	5 (16.7)	2 (6.7)	0.23	3.39 (0.57–20.10)	0.23	2.4 (0.43-13.75)	0.32
Self-reported behavior change							
6 months	12 (40.0)	9 (30.0)	0.54	1.43 (0.46-4.42)	0.54	1.28 (0.31-5.27)	0.74
12 months	17 (56.7)	15 (50.0)	0.61	1.31 (0.47-3.62)	0.61	1.09 (0.35-3.40)	0.88

Abbreviations: CI, confidence interval; GEE, Generalised estimating equation; OR, odds ratio; PPA, Point-prevalence abstinence.

a Adjusted for age, sex, educational attainment, marital status, employment status, years of smoking, and nicotine dependency by the FTND.

Compared with the control group, the intervention group had a significantly higher percentage of reported intention to quit at 6 months (80.1% [21 of 26] vs. 37.9% [11 of 29], *P* = 0.001) and 12 months (80% [20 of 25] vs. 50% [14 of 28], *P* = 0.02). Although the results showed no significant difference in self-reported behavioral change between the two study groups, the proportion of participants reporting a behavioral change was higher in the intervention group at 6 months (Table 4). In particular, the intervention group reported 10% more successful abstinence from at least one health risk behavior at 12 months (Table 4).

**Table 4:** Self-reported behavior change in the intervention and control groups at 6 and 12 months.

	***N*** **(%)**	
**Health-related behavior**	**Intervention group** **(*n* = 30)**	**Control group** **(*n* = 30)**
**6 months**		
Healthy diet	3 (10.0)	2 (6.7)
Regular physical activity	3 (10.0)	4 (13.3)
Reduce alcohol consumption	1 (3.3)	1 (3.3)
Healthy sleep habit	1 (3.3)	1 (3.3)
Smoking reduction or quitting	4 (13.3)	1 (3.3)
**12 months**		
Healthy diet	5 (16.7)	2 (6.7)
Regular physical activity	6 (20.0)	4 (13.3)
Reduce alcohol consumption	2 (6.7)	1 (3.3)
Healthy sleep habit	2 (6.7)	1 (3.3)
Smoking reduction or quitting	5 (16.7)	2 (6.7)

### Process evaluation

The results of process evaluation showed that all the participants satisfied with the content of the brief MI messages. They generally described the messages received as “*supportive,” “encouraging,” “informative,”* and “*useful*.” Participants also appreciated the use of instant messaging to provide them with ongoing professional advice and support in managing their health-related lifestyles. In addition, participants found the assessment methods acceptable and easy to follow, as well as the frequency of brief MI messages appropriate.

## Discussion

This study addresses the important requirement of identifying an innovative strategy to help a ‘hard to reach' group, smokers with NCDs, to quit smoking, improve their health and reduce premature mortality. Consistent with previous studies (6, 8, 9), this study showed that many smokers with NCDs were chronic smokers with a long smoking history, relatively high nicotine dependency with no previous attempts at quitting, and no intention of quitting. The group of chronic smokers represents a challenge because they are less likely to be affected by the current tobacco control interventions or policies (31–33) and will eventually make up a larger proportion of the remaining smokers in the community. Specifically, this research is original and helps to clarify the potential efficacy and feasibility of a general health promotion approach that uses instant messaging to deliver brief MI to help smokers with NCDs quit smoking.

Although the difference in biochemically validated abstinence rates between the intervention and control groups was not statistically significant at 12 months, the results revealed that the abstinence rate of the participants in the intervention group was double that of the participants in the control group (16.7 vs. 6.7%). Furthermore, the biochemically validated abstinence rates in this trial were higher than those in previous trials of different strategies to help smokers with NCDs quit smoking (9–11). However, caution must be taken when interpreting these findings. As this is a pilot study, a small sample size is usually used. For small samples, even very large differences between groups do not become statistically significant. The non-significant difference between the intervention and control groups in our pilot study might be attributable to the relatively small sample size. These findings suggest the requirement of a large RCT in the near future to identify strong evidence to support the use of a general health promotion approach that uses brief MI via instant messaging as a strategy to help smokers with NCDs quit smoking.

Other than potential efficacy, this study suggested the feasibility of this instant messaging-based general health promotion approach in helping smokers with NCDs quit smoking (major objective of this study). Specifically, the results showed that 95% of smokers with NCDs identified in the SOPC owned a smartphone and could use instant messaging applications. Both the response and retention rates of the eligible participants were more than 70%. Instant messaging delivered via mobile devices has been increasingly used and gained support by the WHO for health promotion and treatment compliance (20, 34). The use of such instant messaging applications to deliver brief MI to participants has some advantages; for example, nurses can offer quick, real-time interactions to deliver continuous professional advice and personalized support to patients to help them quit smoking and overcome withdrawal symptoms or cravings. Most importantly, instant messaging through applications is more flexible, efficient and time-saving than face-to-face meetings, particularly during pandemic, where the delivery of face-to-face health care interventions may not be feasible. Direct comparison with other previous trials may not be applicable owing to differences in types of mobile health intervention used, characteristics of target population and theoretical frameworks for study design (35). Nonetheless, the results of this study showed a higher odds of biochemically validated smoking abstinence at 6-month follow-up than that in a Cochrane meta-analysis of 12 trials on mobile health intervention for smoking cessation (21). Future studies could also explore the use of brief MI delivered via different methods for promoting smoking cessation target at different groups of smokers. It is also suggested that more trials should be conducted to further investigate and optimize the use of mobile instant messaging tools in the field of smoking cessation.

This trial had some strengths. We complied with the ‘Russell Standard', which is the gold standard to perform biochemical validation of self-reported abstinence at follow-ups (36). In addition, we conducted data collection in accordance with the guidelines of the Society for Research on Nicotine and Tobacco (27). This ensured the consistency of reporting and allowed direct comparisons of our findings with those of other smoking cessation trials.

This study had several limitations. First, this study used small sample size and the all data were collected in one setting limit the generalizability of the results. Second, owing to the intervention design, participants were eligible only if they were smartphone users and able to use mobile instant messaging tools. The baseline characteristics of those who were excluded due to not meeting this criteria were unable to examine and compare. Future studies could explore and develop other appropriate interventions to promote smoking cessation for this population. Third, this study targeted at smokers with no intention to quit, who might be most likely to reject or did not comply with a smoking cessation intervention. The smoking characteristics of the participants might result in an estimation toward the null value. Nevertheless, the participation rate was 73.2%, which was satisfactory when compared with other smoking cessation trials targeted at patients with NCDs (26).

### Practice implications

The findings of this study provide insights into and evidence of the delivery of brief MI using mobile devices to promote smoking cessation. Unlike a traditional MI, the brief MI took less time to implement, making them more feasible in busy clinical settings. It is anticipated that after minimal training by our smoking cessation team, nurses working at SPOCs can be able to provide initial face-to-face brief MI to smokers with NCDs to motivate them to quit smoking while they are waiting for medical consultation. The nurses can then actively refer smokers to existing cessation services to help them quit smoking through continuous personalized support. A previous study provided evidence that active referral of smokers to existing cessation services could effectively increase cessation (37). Most importantly, the findings can be used to create a new smoking cessation service model that implements a flexible, proactive and personalized approach to help smokers with NCDs quit smoking.

Using a general health approach coupled with brief MI can assist smokers with NCDs to quit smoking and change their unhealthy behaviors simultaneously, eventually improving their physical well-being. Our findings will also inform future research to develop and test a general health promotion approach with brief MI as a strategy to promote healthy lifestyle practices, such as healthy eating, increased physical activity, and reduced alcohol consumption, among individuals with unhealthy behaviors other than smoking, with the aim of preventing and controlling NCDs.

## Conclusions

This study suggested the feasibility and potential efficacy of a general health promotion approach that uses instant messaging to deliver brief MI to help smokers with NCDs quit smoking. Findings from this study support a fully powered RCT of using this innovative strategy to provide rigorous empirical scrutiny of the efficacy of such an approach.

## Data availability statement

The raw data supporting the conclusions of this article will be made available by the authors, without undue reservation.

## Ethics statement

The studies involving human participants were reviewed and approved by the Institutional Review Board of the University of Hong Kong/ Hospital Authority Hong Kong West Cluster. The patients/participants provided their written informed consent to participate in this study.

## Author contributions

WL initiated the study design, analyzed and interpreted the data, and drafted the manuscript. LH, AC, and WX contributed to the study design, analyzed and interpreted the data, and critically revised the manuscript for important intellectual content. MW, DC, and TL provided practical and research knowledge on study implementation. All authors have read and approved the final version of the manuscript.

## Funding

The trial was supported by Health and Medical Research Fund of the Food and Health Bureau, Hong Kong, grant number 16172831. The funding source had no involvement in study design; in the collection, analysis, and interpretation of data; in the writing of the report; and in the decision to submit the article for publication.

## Conflict of interest

The authors declare that the research was conducted in the absence of any commercial or financial relationships that could be construed as a potential conflict of interest.

## Publisher's note

All claims expressed in this article are solely those of the authors and do not necessarily represent those of their affiliated organizations, or those of the publisher, the editors and the reviewers. Any product that may be evaluated in this article, or claim that may be made by its manufacturer, is not guaranteed or endorsed by the publisher.
